# Food-Borne Disease Outbreak of Diarrhetic Shellfish Poisoning Due to Toxic Mussel Consumption: The First Recorded Outbreak in China

**DOI:** 10.1371/journal.pone.0065049

**Published:** 2013-05-21

**Authors:** Tingrui Chen, Xuqing Xu, Jinjiao Wei, Jiang Chen, Renchao Miu, Liming Huang, Xiaoxiao Zhou, Yun Fu, Rui Yan, Zhen Wang, Biyao Liu, Fan He

**Affiliations:** 1 Cangnan Center for Disease Control and Prevention, Cangnan, People's Republic of China; 2 Zhejiang Center for Disease Control and Prevention, Hangzhou, People's Republic of China; 3 Wenzhou Center for Disease Control and Prevention, Wenzhou, People's Republic of China; 4 Hangzhou Center for Disease Control and Prevention, Hangzhou, People's Republic of China; 5 Taizhou Center for Disease Control and Prevention, Taizhou, People's Republic of China; 6 Huzhou Center for Disease Control and Prevention, Huzhou, People's Republic of China; The Australian National University, Australia

## Abstract

**Objectives:**

This investigation was undertaken in response to an outbreak of suspected shellfish poisoning in Zhejiang Province, China. The objectives of this project were to confirm the outbreak and to identify the aetiology, source and mode of transmission.

**Methods:**

A probable case was defined as an individual with diarrhea (≥3 times/day) plus at least one of the following symptoms: fever (≥37.5°C), vomiting, or abdominal pain after consuming seafood between May 23^rd^ and May 28^th^, 2011. Using a case-control study design, we compared exposures to suspected seafood items and cooking methods between 61 probable cases and 61 controls.

**Results:**

Over 220 suspected or probable cases of diarrhetic shellfish poisoning (DSP) were identified (incidence of 18 cases per 100,000). The case control study revealed that 100% of cases and 18% of controls had eaten mussels during the exposure period (OR = ∞, χ^2^ = 84.72,P = 0.000). The number of mussels consumed was related to DSP risk (P = 0.004, χ2 test for trend). Consumption of other seafood items was not associated with disease. The frequency of diarrhea and vomiting were positively correlated with the number of mussels consumed (r = 0.424 and r = 0.562, respectively). The frequency of vomiting and the incubation period were significantly correlated with the total time the mussels were boiled (r = 0.594 and r = −0.336, respectively). Mussels from 3 food markets and one family contained Okadaic acid (OA) and Dinophysistoxin-1 (DTX-1).

**Conclusions:**

This outbreak was attributed to the consumption of mussels contaminated by DSP-toxins (OA and DTX-1) which are produced by different species of dinoflagellates (toxic microalgae) from the genus *Dinophysis* or *Prorocentrum*. Suspension of mussel sales and early public announcements were highly effective in controlling the outbreak, although oversight of seafood quality should be a priority to prevent future contamination and outbreaks.

## Introduction

Shellfish poisoning is a significant public health problem in China [1], [2], [3]. To date five groups of shellfish toxins have been identified: diarrhetic shellfish poisoning (DSP), paralytic shellfish poisoning (PSP), amnesic shellfish poisoning (ASP), neurotoxic shellfish poisoning (NSP) and azaspiracid shellfish poisoning (AZP). DSP in humans is caused by the ingestion of bivalves such as mussels, scallops, oysters or clams contaminated by toxins produced by harmful phytoplankton. Typical DSP symptoms include diarrhea, nausea, vomiting and abdominal pain that manifests 30 minutes to 4 hours after consumption. Patients do not typically present with fever and fully recover without special treatment after several days [4]. The incidence of harmful phytoplankton in China has increased over the past forty years. There were 3 reports in the 1970's, 29 reports in the 1980's, and 40–50 reports in the 1990's. Okadaic acid (OA) and Dinophysistoxin-1 (DTX-1), two toxins associated with DSP, were identified in these harmful phytoplankton along the coast lines of the South and East China Seas [3].

**Figure 1:**
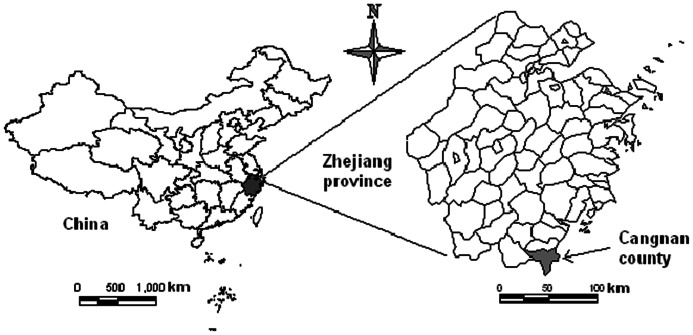
Location of Cangnan county in China.

The wide availability and low cost of shellfish poses a threat to local people and tourists. On May 27, 2011, fifty-seven cases of diarrhea, abdominal pain, and vomiting were reported from three collective units in Cangnan County, Zhejiang province. The symptoms were similar to those caused by DSP. This county lays in the southeast corner of Zhejiang Province, China, which consists of 36 towns and has 1,384,240 residents. It is physically located in the southeast of China, near to the East China Sea. The boundaries of this county lie at longitude 120°7′–121°07′1 and latitude 27°06′–27°36′1, creating 1261.08 square kilometers of land area and 37,200 square kilometers of coastal area. This county has 168.88 kilometers of coastline, which is directly connected to the coastal area of Fujian province, China. (Figure 1).

To our knowledge, this is the first outbreak of DSP in China. There is a dearth of epidemiological knowledge about dose-response factors in DSP. The risk of developing DSP after consuming toxic mussels and the relationship between ingestion and clinical severity are not well understood. The lack of such information hinders the establishment of guidelines for DSP outbreaks. To address this knowledge gap, we conducted an investigation to verify the outbreak and to (1) identify the cause of the outbreak, (2) identify the source of infection and (3) elucidate the mode of transmission. Using a case-control study design, we analyzed the relationship between risk, clinical severity and exposed dose, which will lead to evidence-based guidelines to manage DSP outbreaks in China.

## Materials and Methods

This investigation was undertaken in response to a public health emergency. As such, the investigation was exempt from the requirement for ethical approval and informed consent.

We identified suspected cases of DSP through review of medical records. A suspected case was defined as a resident of Cangnan county who developed one of the following symptoms: diarrhea (≥3 times per day), vomiting, or abdominal pain after consuming seafood between the 23^rd^ and 28^th^ of May 2011. A probable case exhibited diarrhea (≥3 times per day) plus one or more of the following symptoms: fever (≥37.5°C), vomiting, or abdominal pain after consuming seafood between the 23^rd^ and 28^th^ of May 2011.

We identified cases via medical records in all hospitals (550) and private clinics (42) throughout the county. A data collection tool was developed to record the following information: patient's name, age, gender, address, date of onset, date of exposure, diarrhea frequency (per 24 h), vomiting, abdominal pain, fever, tingling, numbness and muscle pain. The county hospitals were responsible for searching cases from their outpatient records, and the township hospitals were asked to search for cases via their own medical records and private clinics.

The most likely cause of the outbreak was from seafood polluted by a shellfish toxin and we carried out this unmatched case control study to test this hypothesis. We included probable cases from the 4 towns that contained the majority of reported cases. Control subjects were the neighbors of the probable cases as long as they presented with no symptoms after the consuming seafood between May 23^rd^ and 28^th^, 2011. The sample size of case and control was computed using the established formula [5]: 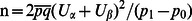, 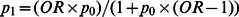, 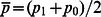, 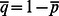. We assumed that the odds ratio (OR) and p_0_ were 5.0 and 0.6, respectively. Given these parameters, the required group sample size was 51.

Nine samples of fresh mussels from 9 local markets, one wild mussel sample, 9 other shellfish and snail from 3 additional markets, and one sample of mussel from a case's home were tested for OA and DTX-1. These samples were tested using Ultra Performance Liquid Chromatography coupled with Triple Quadruple Mass Spectrometry (UPLC-MS/MS) [6] at the Wenzhou Center for Disease Control and Prevention. The calibration standard (Certificate Number: NRC CRM-OA-C) for UPLC-MS/MS were acquired from Institute for Marine Biosciences Canada(NRC).

Age and sex characteristics of cases and controls were compared using a Student's t test and the Pearson chi square test. A Chi square test for trend was also used to analyze dose-response. Spearman correlations were calculated for clinical characteristics (including the frequency of diarrhea and vomiting) and (i) mussel consumption (ii) time for which the mussels were boiled. Statistical analyses were performed with the use of SPSS software (version 15.0). Statistical significance was defined as P<0.05.

## Results

Overall, we found 226 cases (56 suspected, 170 probable) with symptomatic onset between May 25 and 30, 2011. The overall incidence was 18/100,000. Cases were distributed in 23 towns with incidence that range from 1.2 to 102 per 100,000. Eight of the towns had incidence over 50/100,000. The mean age of the cases was 41 years (range: 4 to 85 years) and 50.88% (n = 115) were male. The overall incidences for males and females were 15.88/100,000 and 15.15/100,000, respectively. Commonly reported symptoms included abdominal pain (82%), diarrhea (75%), nausea (72%), vomiting (46%), fever (11%), muscle pain (8.0%), tingling (3.1%), and numbness (2.7%). No case required hospitalization and all patients recovered uneventfully.

**Figure 2:**
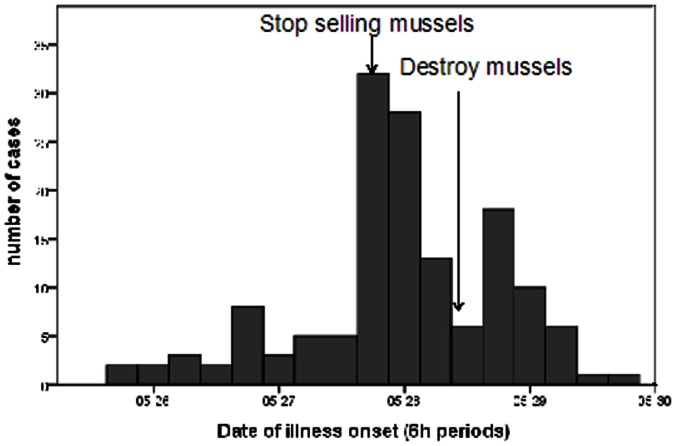
Epidemic curve for reported cases caused by shellfish toxins in Cangnan county, Zhejiang province, China, 2011 (6 h interval).

Ninety-one percent had onset of symptoms within 24 hours of consuming mussels (median: 9.0 hours, range: 30 minutes to 57 hours). The first case occurred at 3:00 PM, May 25^th^. The number of cases increased sharply on the evening of May 27^th^, after which the selling of mussels was prohibited. Following May 27^th^, the number of new cases declined (Figure 2).

The outbreak was traced to seafood polluted by a shellfish toxin given the following information: (1) Almost all interviewed cases ate mussels or other seafood; (2) General heating of the mussels could not effectively remove the pathogen; (3) The number of cases declined sharply after the sale of mussels was prohibited; (4) Harmful phytoplankton (mainly *Prorocentrum donghaiense*) were reported along the coast of Zhejiang and Fujian provinces in May; (5) Similar cases and symptoms were reported after consumption of seafood in Ningbo and Zhoushan, two cities of Zhejiang Province.

There were 61 cases and 61 controls enrolled in the case-control study. The mean ages of cases and controls were 40.47 ± 13.50y and 36.88 ± 11.36y, respectively, (t = 1.589, P = 0.115). The gender ratio did not differ significantly between cases (28 males and 33 females) and controls (28 males and 33 females) (χ2 = 0.295, P = 0.587).

Between May 23^rd^ and 28^th^, 100% of the cases had eaten mussels compared to 18% of the controls (P = 0.000). Dose response analysis showed that the OR increased with the number of mussels consumed (trend χ2 = 5.31, P = 0.021). Other seafood, including fish, crab, snail and shrimp showed either an inverse association with illness or no association at all (Table 1 and 2).

**Table 1:** Odds ratios for selected seafood in a food-borne disease outbreak, Cangnan county, Zhejiang province, China 2011.

Seafood eaten	N		%		OR 95%CI	Pvalues
	cases	controls	cases	controls		
mussel	61	11	100	18	∞*	0.000
fish	9	27	15	44	0.22(0.091–0.52)	0.001
crab	5	21	8.2	34	0.17(0.059–0.49)	0.001
snail	5	14	8.2	23	0.30(0.10–0.89)	0.044
shrimp	2	3	3.3	4.9	0.66(0.11–4.1)	1.000

* Chi-square for trend: χ2 = 84.72, P = 0.000.

**Table 2:** Odds ratios for pieces of mussel eaten in a food-borne disease outbreak, Cangnan county, Zhejiang province, China 2011.

The number of pieces of mussel	N		%		OR 95%CI	P-values
	case	control	case	control		
≤5	10	6	17	60	Ref	
6–15	40	4	67	40	6.0(1.4–25)	0.017
≥16	10	0	17	0	∞	0.053

* Chi-square for trend: χ2 = 8.354,P = 0.004.

**Figure 3:**
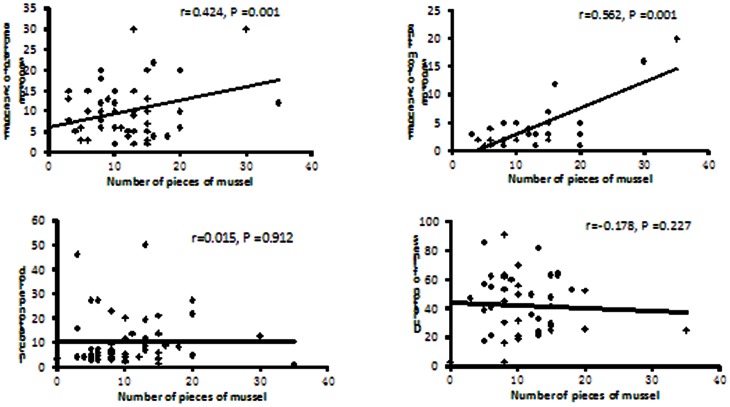
The correlation of clinical characteristics with number of pieces of mussel eaten in a food-borne disease outbreak, Cangnan county, Zhejiang province, China, 2011.

**Figure 4:**
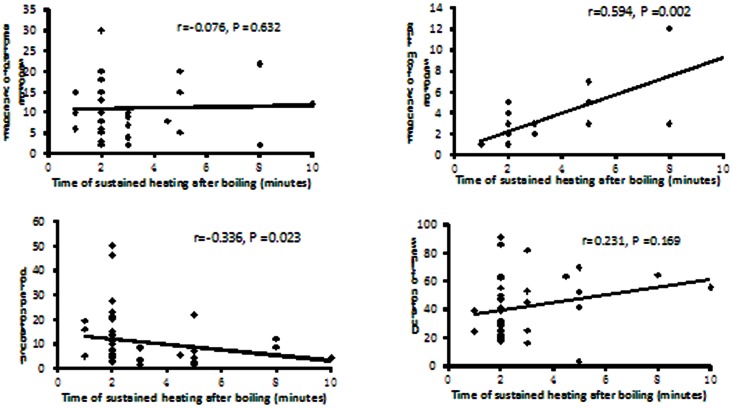
The correlation of clinical characteristics with the total time mussels were boiled in a food-borne disease outbreak, Cangnan county, Zhejiang province, China, 2011.

The frequency of diarrhea and vomiting were postively correlated with the number of mussels consumed, showing correlation coefficients of 0.424 and 0.562, respectively (Figure 3). The frequency of vomiting was significantly correlated with the total amount of time the mussels were boiled (r = 0.594, P = 0.002). Moreover, the incubation period was negatively correlated with the total amount of time the mussels were boiled (r = −0.336, P = 0.023). (Figure 4).

Three of nine fresh mussels from local markets and one leftover mussel from a case's home were positive for OA (157 µg/kg, 349 µg/kg, 611 µg/kg and 635 µg/kg) or DTX-1 (16 µg/kg, 48 µg/kg, 48 µg/kg and 86 µg/kg). The detection limit for OA or DTX-1 is 10 µg/kg. The 9 other fresh shellfish and snails purchased from multiple local suppliers in 3 different markets were negative for OA and DTX-1.

We reviewed and analyzed the sales records of all sellers to track the sources of contaminated mussels. We found that the local mussels did not come into the market and the contaminated mussels may have come from the Lianjiang county of Fujian province.

## Discussion

This investigation revealed an outbreak of food-borne disease in Cangnan county was caused by DSP toxins. This pathogen was transmitted via the consumption of mussels contaminated by toxic phytoplankton.

Our results agree with previous work that found symptom frequency and intensity, including that for diarrhea and vomiting, were positively correlated with the number of mussels consumed[4], while there was no correlation between the number of mussels consumed and the incubation period or the duration of illness.

The DSP toxins are all heat-stable polyether and lipophilic compounds isolated from various species of shellfish and dinoflagellates [7]. There are currently no useful methods available for effectively reducing phytotoxins in contaminated shellfish as denaturing of these toxins only occurs after prolonged boiling (163 minutes) at 100°C [8]. Cooking does not alter the toxicity of the contaminated shellfish, but intoxication can be avoided if the digestive glands were removed before preparation [9]. However, many persons interviewed during this investigation reported that they left the digestive glands attached to mussels and the mussels were consumed in a soup. In this study, sustained heating did not decrease the risk of DSP and did not reduce the symptoms, including total frequency of diarrhea and total duration of symptoms. In fact, increased boiling time was associated with increased vomiting and a shortened incubation period. This may be a result of the toxins being released into the soup as the mussels were boiled.

In light of the findings from this investigation, we recommended that selling of mussels be temporarily stopped. On May 28^th^, the food security office of Zhejiang province raised the warning and suggested the public not eat mussels. All leftover mussels were destroyed. After 2 days the number of cases declined sharply. However, after the sale of mussels was prohibited and 750 Kg of mussels were destroyed in Zhejiang province, the sellers were forced to sell mussels in the Fujian province. This was a likely cause of the outbreak in the Fujian province on April 29^th^. Unfortunately, further epidemiological investigations into the source of contaminated mussels were not conducted.

Similar to Hong Kong [10], China has no rigorous monitoring programs for shellfish. Public awareness and education were the best alternative to prevent and control the outbreak. According to the results of this study, eliminating the digestive glands of mussels before cooking and frying, rather than boiling, the mussels may reduce the likelihood of intoxication. Monitoring and supervision of seafood quality should be established in the immediate future to prevent further outbreaks.

In summary, contaminated mussels, likely as a result of recent harmful phytoplankton blooms, were the underlying cause of the DSP outbreak. The suspension of mussel sales and early public announcements were highly effective in controlling the outbreak, although oversight of seafood quality should be a priority to prevent future contamination and outbreaks.

## References

[1] Anderson DM, Kulis DM, Qi YZ, Zheng L, Lu S, et al (1996) Paralytic shellfish poisoning in southern China. Toxicon 34: 579–590. 8783452 10.1016/0041-0101(95)00158-1

[2] Wang ZH, Nie XP, Jiang SJ, Zhao JG, Cao Y, et al (2011) Source and profile of paralytic shellfish poisoning toxins in shellfish in Daya Bay, South China Sea. Mar Environ Res 72: 53–59. 21658755 10.1016/j.marenvres.2011.04.007

[3] Zhou M, Li J, Luckas B, Yu R, Yan T, et al (1999) A Recent Shellfish Toxin Investigation in China. Mar Poll Bull 39: 331–334.

[4] Fleming LE. Diarrhetic Shellfish Poisoning. Available: http://www.whoi.edu/redtide/page.do?pid=9679&tid=523&cid=27688. Accessed 2012 Dec 23.

[5] Li LM (2004) Epidemiology. 5th ed.Beijing:People's medical publishing house. 90 p.

[6] Fux E, McMillan D, Bire R, Hess P (2007) Development of an ultra-performance liquid chromatography-mass spectrometry method for the detection of lipophilic marine toxins. J Chromatogr A 1157: 273–280. 17521661 10.1016/j.chroma.2007.05.016

[7] Draisci R, Lucentini L, Giannetti L, Boria P, Poletti R (1996) First report of pectenotoxin-2 (PTX-2) in algae (Dinophysis fortii) related to seafood poisoning in Europe. Toxicon 34: 923–935. 8875779 10.1016/0041-0101(96)00030-x

[8] Scoging AC (1991) Illness associated with seafood. Communicable Disease Report 1: 117–128.

[9] Viviani R (1992) Eutrophication, marine biotoxins, human health. Sci Total Environ Suppl: 631–662. 10.1016/b978-0-444-89990-3.50056-0 1475681

[10] Chung PH, Chuang SK, Tsang T (2006) Consumption of viscera as the most important risk factor in the largest outbreak of shellfish poisoning in Hong Kong, 2005. Southeast Asian J Trop Med Public Health 37: 120–125. 16771223

